# Metagenomics Analysis of the Wheat Virome Identifies Novel Plant and Fungal-Associated Viral Sequences

**DOI:** 10.3390/v13122457

**Published:** 2021-12-07

**Authors:** Carla Dizon Redila, Ved Prakash, Shahideh Nouri

**Affiliations:** Department of Plant Pathology, Kansas State University, Manhattan, KS 66506, USA; cbdizon@ksu.edu (C.D.R.); vedp@ksu.edu (V.P.)

**Keywords:** wheat virome, metagenomics, RNA-seq, umbra-like virus, vipovirus, wheat fungal-associated viruses

## Abstract

Wheat viruses including wheat streak mosaic virus, Triticum mosaic virus, and barley yellow dwarf virus cost substantial losses in crop yields every year. Although there have been extensive studies conducted on these known wheat viruses, currently, there is limited knowledge about all components of the wheat (*Triticum aestivum* L.) virome. Here, we determined the composition of the wheat virome through total RNA deep sequencing of field-collected leaf samples. Sequences were de novo assembled after removing the host reads, and BLASTx searches were conducted. In addition to the documented wheat viruses, novel plant and fungal-associated viral sequences were identified. We obtained the full genome sequence of the first umbra-like associated RNA virus tentatively named wheat umbra-like virus in cereals. Moreover, a novel bi-segmented putative virus tentatively named wheat-associated vipovirus sharing low but significant similarity with both plant and fungal-associated viruses was identified. Additionally, a new putative fungal-associated tobamo-like virus and novel putative *Mitovirus* were discovered in wheat samples. The discovery and characterization of novel viral sequences associated with wheat is important to determine if these putative viruses may pose a threat to the wheat industry or have the potential to be used as new biological control agents for wheat pathogens either as wild-type or recombinant viruses.

## 1. Introduction

Wheat (*Triticum aestivum* L.) is one of the leading staple crops in the world; however, wheat viral diseases continue to pose a great threat to the industry [1]. Wheat viruses including wheat streak mosaic virus (WSMV), Triticum mosaic virus (TriMV), High Plains wheat mosaic emaravirus (HPWMoV), soilborne wheat mosaic virus (SBWMV), barley yellow dwarf virus (BYDV), and cereal yellow dwarf virus (CYDV) have a significant impact in the Great Plains and other wheat growing regions around the world [2,3,4]. WSMV, TriMV, and HPWMoV have been reported as causal agents of the wheat streak mosaic disease (WSM) complex; a destructive disease-causing significant yield loss [5]. In 2017, WSM caused a total of $76 million in yield loss to Kansas farmers [5]. WSMV and TriMV are type species classified under the *Potyviridae* family and are both filamentous viruses with positive-sense, single-stranded RNA genomes [6,7,8]. In contrast, HPWMoV belongs to the *Fimoviridae* family, which is a multipartite, negative-sense virus consisting of 8 RNA segments [9]. All 3 viruses are naturally transmitted by the wheat curl mite (WCM), *Aceria tosichella* Kiefer [10]. The typical symptoms of WSM are yellow, mosaic-like streaks on the leaves which lead to chlorosis and reduction in photosynthetic capabilities. Severe infection may also lead to stunted growth.

BYDV and CYDV are other economically important known wheat viruses from the family *Luteoviridae,* genera *Luteovirus* and *Polerovirus,* respectively. BYDV and CYDV are causal agents of the barley yellow dwarf disease (BYD) [11,12] transmitted mainly by the bird cherry-oat aphid (*Rhopalosiphum padi*) [13]. Stunting and discoloration of leaves are typical symptoms of BYD. Historically, the 20-year average yield loss caused by BYD is 1% in Kansas; however, these losses can increase up to 49% when conditions are conducive for the proliferation of *R. padi*, the natural vector of BYD viruses [14,15].

Despite successful breeding programs, wheat viral diseases are still major issues for farmers. Enormous studies have been conducted to characterize these wheat pathogenic viruses and determine virus-host interactions [16,17]. However, most of these studies have focused on the documented wheat viruses [2,4], and there has been no metagenomics study to analyze all viral communities associated with wheat.

Viruses are the most abundant habitants of the earth, infecting both prokaryotes and eukaryotes [18,19,20]. The study of virology has been spurred on by the identification of a plant virus, tobacco mosaic virus, which was the first virus to be discovered in 1898 [21]. Since then, a multitude of virology studies have been conducted to identify and characterize pathogenic viruses causing disease in humans, animals, and plants [22,23] and determine the relationships of viral pathogens with their hosts and vectors [24,25,26]. With the introduction of high-throughput next-generation sequencing (NGS) and advanced bioinformatics tools, a new age of virus discovery has commenced in the last decade and many novel pathogenic and non-pathogenic viruses have been identified [27,28]. Indeed, studying viral communities associated with a particular organism, ecosystem, or holobiont (virome) in the metagenomics era has been a significant contribution to the field of virology, including plant virology [29,30]. The identification of persistent viruses which may be potential biological control candidates [31,32], the discovery of novel and newly emerging viruses [31,33,34], and the determining of evolutionary relationships of economically important viral pathogens [35,36] are some of these contributions.

To address the current knowledge gap about the wheat virome, we conducted a metagenomics study with field-collected wheat leaf samples. Through high-throughput total RNA sequencing, not only known wheat viruses but novel plant and fungal-associated viral sequences were also identified in this study. The discovery and characterization of new viruses is crucial due to the fact that these viruses may be emerging viral pathogens and pose a threat to the wheat industry or be beneficial and have potential to serve as new biological controls for wheat pathogens either as wild-type or recombinant viruses.

## 2. Materials and Methods

### 2.1. Sample Collection

Symptomatic wheat leaf samples were collected in 2019 and 2020 from Kansas fields. In addition to surveyed samples, samples sent to the Kansas State University Plant Disease Diagnostics Lab and the provided field samples from Nebraska, Montana, and Colorado fields were also added to the collection. A total of 30 samples were chosen for total RNA library preparation and sequencing based on the geographic locations (Table S1).

### 2.2. RNA Extraction and Library Preparation

Total RNAs from 100 mg of wheat leaf tissues were extracted using TRIzol reagent (Invitrogen, CA, USA), according to the manufacturer’s instruction. The extracted RNAs were treated with DNase I (Zymo Research, CA, USA). The integrity and quantity of the DNase-treated RNAs were measured using Qubit 4 (Invitrogen, CA, USA) with the RNA IQ assay kit. The TruSeq Stranded Total RNA with Ribo-Zero Plant kit (Illumina Inc., CA, USA) was utilized to remove the rRNA and prepare the libraries for sequencing following the manufacturer’s instruction. Agencourt RNAClean XP (Beckman Coulter, MA, USA) was used to purify the samples to ensure the removal of all traces of rRNA. TruSeq RNA Single Indexes Sets A and B (Illumina Inc., CA, USA) were used for adapter ligation. After each step of cDNA synthesis, adapter ligation, and enrichment of the DNA fragments, the samples were purified using the Agencourt AMPure XP (Beckman Coulter, MA, USA). The final libraries were subjected to quality control analysis using Agilent Bioanalyzer 2100 system (Agilent Technologies, CA, USA) and were quantified using the Qubit 4 (Invitrogen, CA, USA) with the 1X dsDNA High Sensitivity Assay (Invitrogen, CA, USA). At the Kansas State Integrated Genomics Facility, cDNA libraries were indexed using unique identifiers, pooled (2 sets of 15) and sequenced in two lanes using the NextSeq 500 platform for high-output with a read length of 1 × 75 bp.

### 2.3. RNASeq Analysis and Virus Genome Identification

Trimmomatic was used to trim the reads for quality, length, and the adapter sequences [37]. To ensure the reads no longer contained adapter sequences and were of high quality, FastQC was utilized for quality control [38]. STAR Aligner was used to index the wheat reference genome obtained from Ensemble Genomes project and align the indexed reference to the reads [39,40]. The unmapped reads were extracted and used for mapping and de novo assembly using two assemblers: CLC Genomics Workbench 20 (CLC Bio, Qiagen, MD, USA) and Trinity 2.8.0 [41]. All de novo assembled contigs were used as queries for BLASTx searches against the non-redundant protein database with the search parameter limited to the virus taxids and E-value < 0.001 [42].

### 2.4. Viral Genome Sequence Validation

To validate the identified viral sequences, the first strand cDNAs were synthesized using original RNAs and SuperScript II Reverse Transcriptase (Invitrogen, CA, USA) with oligo dT and /or gene specific primers designed using the de novo-assembled contigs. For PCR, the 25 µL reaction contained 1x GoTaq Flexi Buffer (Promega, WI, USA), 1 µM MgCl2, 0.1 µM dNTP, 0.4 µM of gene specific primers (Table S2), 1.25 U GoTaq Flexi DNA Polymerase (Promega, WI, USA), and nuclease-free water. The thermal cycle program for the GoTaq Flexi protocol is as follows: 94 °C for 2 min, 34 cycles of 94 °C for 10 s, 55 °C for 15 s, 72 °C for 2 min, and 72 °C for 5 min. The PCR products were cleaned using the DNA Gel Extraction Kit (Zymo Research, CA, USA) and sequences confirmed through Sanger sequencing.

### 2.5. Rapid Amplification of cDNA Ends

The 5′ and 3′ untranslated regions (UTRs) of the novel viral sequences were completed through rapid amplification of cDNA ends (RACE) using gene specific primers and the SMARTer RACE 5′/3′ Kit (Takara Bio, CA, USA), following the manufacturer’s instruction. PCR products were cloned using the Zero Blunt II TOPO kit (Invitrogen, CA, USA) and transformed using One Shot TOP10 Chemically Competent *E. coli* (Invitrogen, CA, USA). At least 15 clones for each viral sequence were sent for Sanger sequencing (Genewiz Inc., NJ, USA). Sanger sequencing results were aligned, and consensus sequences were extracted for individual viral sequences.

### 2.6. Small RNA Deep Sequencing

The total RNA of the sample NS02_19 was utilized for small RNA deep sequencing. Total RNA was extracted using mirVana miRNA isolation kit (Thermo Fisher Scientific, CA, USA) and treated with DNase Ι (Zymo Research, CA, USA). The RNA integrity was measured using the Qubit 4 RNA IQ assay (Invitrogen, CA, USA). Two µg of the total RNA was sent to the Beijing Genomics Institute (BGI), Hong Kong for library construction and 50-bp single-read sequencing using the DNBSeq platform.

### 2.7. Phylogenetic Analysis

The phylogenetic analysis was conducted only with novel viral sequences identified in this study. The amino acid sequences were aligned using MUSCLE [43] with the top viral reference sequences showing the highest similarities in BLASTx searches. MEGA 5 was utilized to determine the best substitution model for the alignments [44]. The maximum-likelihood trees were constructed using the PHYML plugin integrated in the Geneious Prime 2020 20.4 program (http://www.geneious.com, accessed 20 June 2020) using the default settings with 1000 bootstrap replicates [45].

## 3. Results

### 3.1. RNASeq Analysis

**Figure 1:**
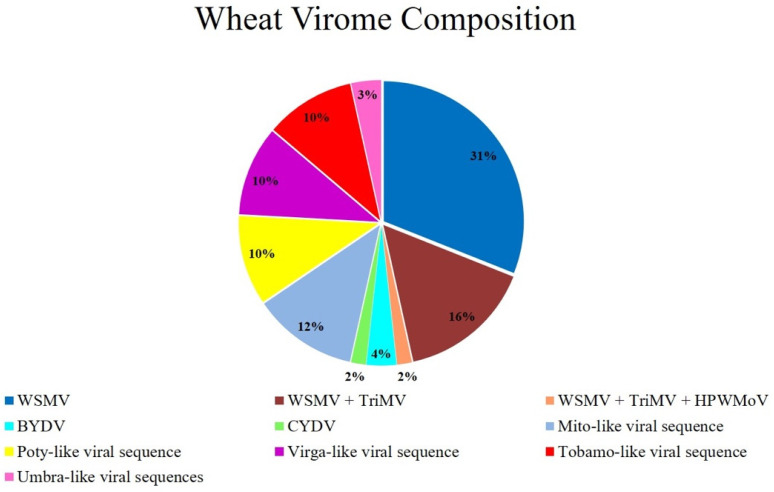
The composition of the virome of the studied wheat samples. Wheat viral communities identified here included known (WSMV, TriMV, HPWMoV, BYDV, CYDV) and novel (umbra-like, virga-like, poty-like, tobamo-like, mito-like) viral sequences.

A total of 980 M reads with an average of 33 M reads per library were obtained (Table S3). Reads are publicly available at NCBI Sequence Read Archive (SRA) under the BioProject number PRJNA722004. Clean reads were mapped into the genome of the wheat (GenBank assembly accession: GCA_900519105.1) [39] and unmapped reads were collected for mapping into the genome of the known wheat viruses (Table S4). After subtracting the mapped reads for known wheat viruses, the remaining reads were de novo assembled and used for BLASTx searches. Figure 1 shows all identified viral sequences by BLASTx searches using contigs generated either by mapping or de novo assembly.

### 3.2. Wheat Virome Composition and Characterizing Novel Viral Sequences

#### 3.2.1. Known Wheat Viruses

The five documented wheat viruses including WSMV, TriMV, HPWMoV, BYDV-PAV, and CYDV were identified (Figure 1) and their full genome sequences were obtained and deposited in the GenBank (Table S5). Full genome sequences of the identified WSM-associated viruses including WSMV, TriMV, and HPWMoV and their accession numbers can be found in the recent publication [46]. WSMV single infections were the most prevalent in the leaf samples among the known wheat viruses. Other known wheat viruses, such as BYDV-PAV and CYDV occurred in low incidences of ~7% (2 out of 30 libraries) and 4% (1 out of 30 libraries), respectively. The presence of these known wheat viral sequences was validated in original RNAs via RT-PCR and Sanger sequencing (Figure S1).

#### 3.2.2. “Umbra-Like” Viral Sequences

BLASTx searches identified contigs of ~1.5 kb in two of our libraries and illustrated ≤ 60% significant (E value < 0.001) similarities to unclassified umbra-like viruses (Table 1). The presence of this viral sequence was confirmed in original RNAs (Figure S1). Both positive wheat samples for the umbra-like viral sequence were from fields located in the same county but in two different years (Table S1).

**Figure 2:**
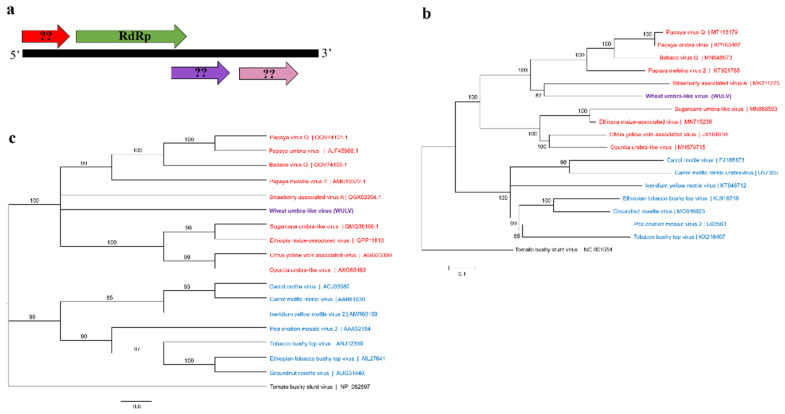
The schematic genome organization of wheat umbra-like virus (WULV) (**a**) and the phylogenetic trees constructed based on the RdRp nucleotide (**b**) and amino acid (**c**) sequences. The genome contains 4 predicted ORFs: ORF1 (viral methyltransferase and viral helicase), ORF2 (RdRp), ORF3 (DEAD-like helicase), and ORF4 (unknown). The PHYML trees were constructed using the substitution methods: T92 + G + I (**b**) and rtREV + G+ I + F (**c**) with a bootstrap value of 1000. The umbra-like associated RNAs (ulaRNAs) are in red and the plant umbraviruses are written in blue text. Tomato bushy stunt virus was used as the outgroup.

The complete nucleotide sequence including the nucleotide sequences of both ends of the genomic RNA of this newly identified umbra-like virus tentatively named wheat umbra-like virus (WULV) was determined by RACE. The full genome size of WULV was 3527 nucleotides with a short 5′UTR of 10 nt and a longer 3′UTR of 237 nt. Four predicted open reading frames (ORF) were determined for WULV using the ORFfinder (https://www.ncbi.nlm.nih.gov/orffinder/, accessed 20 September 2021) (Figure 2a). The predicted ORFs were used as queries to search against the Conserved Domain Database (CDD). ORF1 (nt 11 to 544) encoded a putative protein of 177 aa which did not show any similarity to known proteins at the database. The second ORF, ORF2 (nt 624 to 2098) encoded a large putative protein of 490 aa with the RNA-dependent RNA polymerases (RdRp) conserved domain (Figure 2a). ORF1 and ORF2 are non-overlapping with 80 nt-long intergenic region. ORF3 (nt 1988 to 2626) which has an overlap with ORF2 encoded a putative protein of 212 aa, and additionally ORF4 (nt 2675 to 3295) encoded a putative protein of 206 aa (Figure 2a). A 48 nt-long intergenic region is between ORF3 and ORF4. Neither ORF3 nor ORF4 amino acid sequences demonstrated significant similarities to known proteins in databases.

The RdRp nucleotide sequence of WULV showed the highest identity (65%) and 50% coverage to strawberry associated virus A (MK21275). Phylogenetic trees constructed based on both the RdRp nucleotide and amino acid sequences illustrated a close phylogenetic relationship between WULV and recently identified and reported umbra-like associated RNAs (ulaRNAs) in strawberry, papaya, and babaco (Figure 2b,c). WULV was clustered with strawberry associated virus A based on the RdRp nucleotide sequence, but the amino acid sequence did not place it in a defined clade.

#### 3.2.3. “Poty-Like” and “Virga-Like” Viral Sequences

Contigs with BLASTx hits of ≤25% significant similarity (E value < 0.001) with potyviruses such as barley mild mosaic virus (BaMMV) were found in several libraries (Table 1). Additionally, other viral sequences showing ≤30% significant similarity to recently discovered unclassified virga-like viruses associated with both plant and fungi were found in the same libraries as the poty-like viral sequences (Table 1). Both viral sequences were validated in the original RNAs via RT-PCT and Sanger sequencing (Figure S1).

Two larger individual contigs of ~2200 and 3500 nt were generated through the re-assembly of smaller contigs for poty-like and virga-like sequences, respectively. Discovery of these 2 viral sequences in the same libraries along with their low-level amino acid similarities to newly discovered unclassified viruses belonging to the *Virgaviridae* and also viruses from the family *Potyviridae* suggested that they may potentially belong to a novel putative virus.

The nucleotide sequences of the ends of the genomic RNAs of both poty-like and virga-like sequences were determined by RACE. The complete genome of the virga-like RNA, which contains the 5′ and 3′ UTRs of 107 and 46 nt, respectively, was 3739 nucleotides. The full genome sequence of the poty-like RNA was 2439 nucleotides with the 5′ and 3′ UTRs of 100 and 114 nt, respectively. Both RNAs were polyadenylated. The alignment of the 5′ and 3′ UTRs of these novel viral sequences resulted in an 85% and 45% similarity, respectively (Figure S2). This high level of similarity at the 5′ and 3′ UTRs of the two RNAs strongly suggests that these are two viral segments of one putative novel virus tentatively named wheat-associated vipovirus (WaVPV) with the virga-like and poty-like sequences as RNA1 and RNA2, respectively.

**Figure 3:**
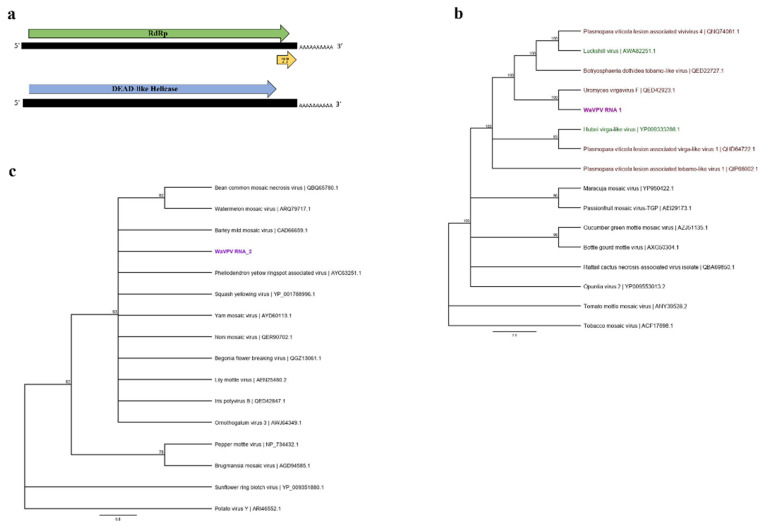
The schematic genome organization of wheat associated vipovirus (WaVPV) (**a**) and the phylogenetic trees constructed based on the RdRp (RNA 1) (**b**) and the DEAD-like helicase (RNA2) (**c**) amino acid sequences. RNA1 has two predicted ORFs: ORF1 containing conserved domain of the RdRp and a small unknown ORF2 towards the 3′UTR. RNA2 contains an ORF corresponding to the DEAD-like helicase. The PHYML trees were constructed using the substitution methods: rtREV + I + G (**b**) and WAG + G (**c**) with a bootstrap value of 1000. Viral sequences used to generate phylogenetic trees were selected from the hits and showed the highest similarity to WaVPV RNA1 and RNA2 in our BLASTx searches. WaVPV RNA1 and RNA2 identified in this study are defined in purple. Fungal-associated viral sequences are written in brown, insect-associated viral sequences in green, and plant viruses are in black text.

Two predicted overlapping ORFs were determined for the WaVPV RNA1 (Figure 3a). ORF1 (nt 108 to 3615) and a small ORF2 (nt 3412 to 3664) encoded putative proteins of 1168 and 83 amino acids, respectively. The predicted ORFs were used as queries to search against the CDD. The ORF1 of RNA1 showed a significant similarity (E-value < 0.0001) with the viral RdRp and contained a conserved GDD motif, while no significant similarity was found for the RNA1-ORF2 deduced amino acid sequence among the known protein sequences in databases (Figure 3a). The WaVPV RNA2 encoded a putative protein of 729 amino acids from a single predicted ORF (nt 101 to 2291) (Figure 3a). Our search using the RNA2 predicted ORF as the query identified a DEAD-like helicase domain with a significant (E-value < 0.0001) similarity (Figure 3a).

The phylogenetic analysis of the amino acid sequence of the ORF1 (RdRp) of WaVPV RNA1 grouped this putative virus with a fungal-associated virus, uromyces virgavirus F, and it is found in a clade of other recently discovered fungal-associated and insect-specific virga-like viruses belonging in the *Virgaviridae* family (Figure 3b). On the other hand, the phylogenetic tree built based on the RNA2 polyprotein grouped WaVPV RNA2 with plant viruses from the *Potyviridae* family (Figure 3c), which supports the BLASTx results.

#### 3.2.4. “Tobamo-Like” Viral Sequences

**Figure 4:**
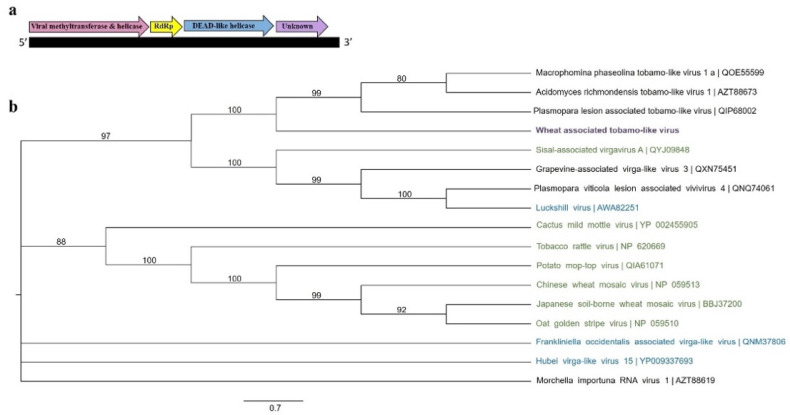
The schematic genome organization of wheat associated tobamo-like virus (WaTBLV) (**a**) and the maximum likelihood tree (**b**) of the amino acid sequence of ORF 2 (RdRp). Four ORFs were predicted for WaTBLV with conserved motifs for viral methyltransferase and viral helicase (ORF1), RdRp (ORF2), and DEAD-like helicase (ORF3). No conserved domain was determined for ORF4. The tree was constructed using the substitution method: GTR + F with a bootstrap value of 1000. Wheat associated tobamo-like virus identified in this study (purple), fungal-associated tobamo-like viruses (black), insect-associated tobamoviruses (blue), and plant tobamoviruses (green) have been defined. The tree did not contain a defined outgroup due to the lack of sufficient information about the taxonomy of this group of viruses.

BLASTX searches also identified contigs with the size range of 2–4 kb in several libraries (Table 1). These results illustrated low (≤40%) but significant (E value < 0.001) amino acid similarities to tobamoviruses including plant, fungal, and insect-associated tobamaviruses. The presence of this novel viral sequence was confirmed in RNA samples via RT-PCR and Sanger sequencing (Figure S1). We were able to generate a nearly complete genome sequence of this novel tobamo-like viral sequence tentatively named wheat associated tobamo-like virus (WaTBLV) through the re-assembly, RACE, and Sanger sequencing. The near complete genome sequence of WaTBLV has a length of 10,285 nt encoding 4 predicted ORFs (Figure 4a). ORF1 encoded a putative large protein of 1647 aa and showed a significant similarity to viral methyltransferase and helicase. ORF2 encoded a putative protein of 400 aa containing an RdRp conserved domain (Figure 4a). ORF3 and ORF4 encoded putative proteins of 859 and 305 aa, respectively. A DEAD-like helicase domain was identified for ORF3; while no conserved domain was found for ORF4.

The phylogenetic analysis of the RdRp amino acid sequence placed WaTBLV in a single sister taxa with other newly identified fungal-associated tobamo-like viruses such as macrophomina phaseolina tobamo-like virus 1 (QOE55599) and plasmopara viticola lesion associated tobamo-like virus 1 (QIP68002) (Figure 4b).

#### 3.2.5. “Mito-Like” Viral Sequences

**Figure 5:**
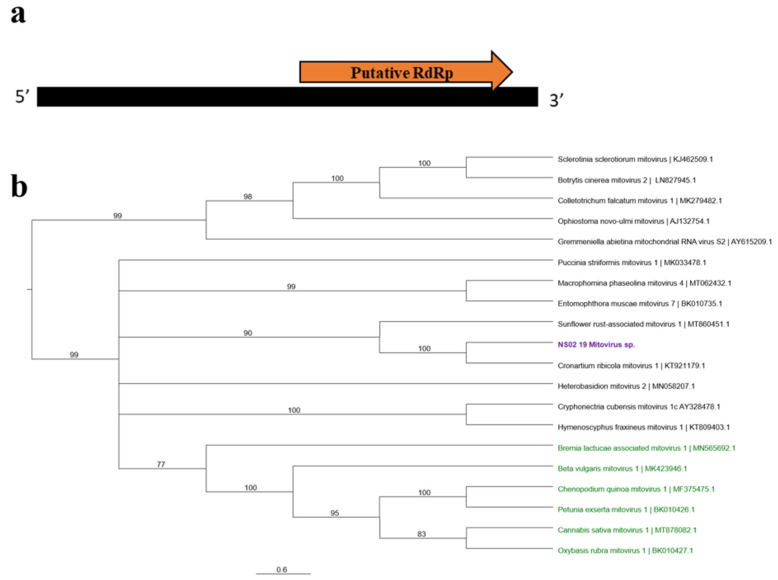
The schematic genome organization (**a**) and the phylogenetic tree of *Mitovirus* sp. (**b**). The genome contains a single ORF with the RdRp conserved domain. The PHYML tree was constructed based on the nucleotide sequence of the full genome using the substitution method: GTR + I + G with a bootstrap value of 1000. The viral sequences used in this analysis included fungal-associated mitoviruses (black) and plant-associated mitoviruses (green). *Mitovirus* sp. is defined in purple text.

BLASTx searches identified several viral sequences showing similarities to mitoviruses (Table 1). The identified mitovirus sequences in our libraries shared low amino acid similarities (~40%) and a query coverage ranging from 70–80% with mitovirus reference sequences affecting rust fungi, suggesting that this putative mitovirus tentatively named *Mitovirus* sp. is novel. We were able to assemble the near complete genome sequence (2642 nt) of *Mitovirus* sp. The genome of *Mitovirus* sp. contains a single ORF demonstrating a significant similarity to the viral RdRp (Figure 5a).

Our phylogenetic analysis using the nucleotide sequence of the near complete genome placed *Mitovirus* sp. in a clade with fungal-associated mitoviruses infecting rust such as cronartium ribicola mitovirus 1 (KT921179) and sunflower rust-associated mitovirus (MT860451) and grouped them away from plant-associated mitoviruses (Figure 5b).

### 3.3. Small RNA Sequencing

**Figure 6:**
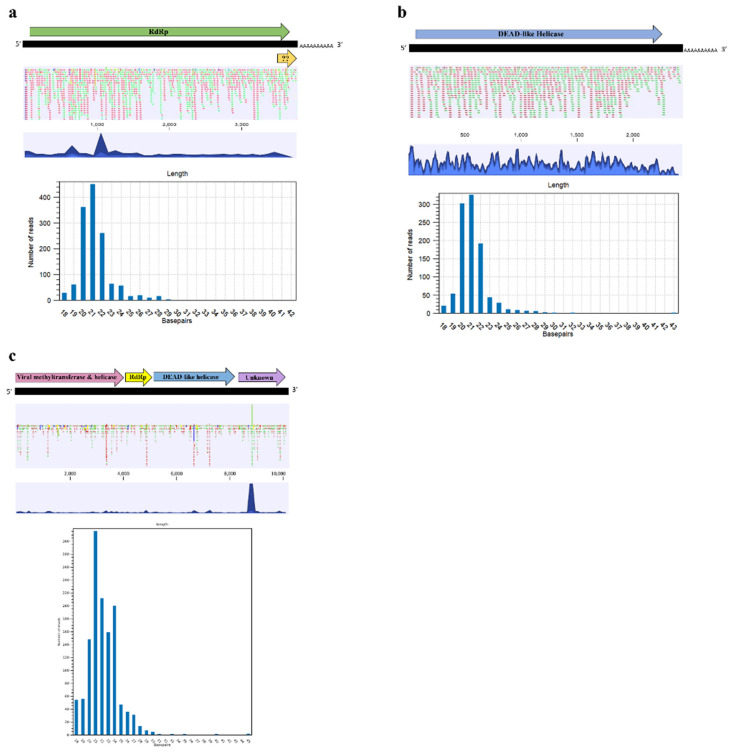
The coverage and size distribution of small RNAs mapped to WaVPV RNA 1 (**a**), RNA 2 (**b**), and WaTBLV (**c**). The small RNA mapping showed strong peaks at the 21 nt for both WaVPV RNAs followed by peaks at the 20 nt. The coverage of the mapping using the full genome of viral sequences was strong throughout the RNA 2 of WaVPV, but two defined peaks were observed for RNA1. Mapping using WaTBLV genome generated small RNAs with a strong peak at the 21 nt followed by a peak at the 24 nt. A single peak located in WaTBLV ORF4 was determined.

To determine if new putative viruses, WaVPV and WaTBLV, are replicative viruses triggering host immune response, we generated the small RNA profile of a positive sample with both novel viral sequences. The small RNA deep sequencing yielded 52 M reads with the length ranging from 15–45 nt. After quality filtering and adapter trimming, the clean reads used for mapping was ~7 M. The clean reads were mapped to the complete genome of WaVPV and produced 1354 and 1007 reads for RNAs 1 and 2, respectively (Figure 6a,b). The size distribution of siRNAs mapped into both RNAs had a prominent peak at 21 nt followed by a peak at 20 nt (Figure 6a,b) suggesting that WaVPV is probably a replicative virus and triggers RNA interference (RNAi) defense mechanism. For the WaVPV RNA1, two mapping hotspots were determined in the genome (Figure 6a), whereas the mapping hotspots for RNA2 was well dispersed throughout the genome (Figure 6b).

By using the near complete genome sequence of WaTBLV as a reference for mapping, 1291 reads with a prominent peak at 21 nt followed by a peak at 24 nt were generated (Figure 6c). This result also suggested that WaTBLV is probably a replicative virus triggering the RNAi antiviral defense mechanism of the host. A single mapping hotspot located in ORF4 was determined in the genome of WaTBLV (Figure 6c).

## 4. Discussion

Wheat documented viruses continue to present a great threat to the wheat industry and global food security. To identify potential undocumented/new putative viruses naturally associated with this important crop which may either be a new threat to the wheat industry or have the potential to be used as new biological agents for the control of wheat pathogens, we determined the composition of the wheat virome using field samples through high throughput sequencing.

Our study was able to uncover that among all known wheat viruses, WSMV is the most dominant virus in the field. The full genome sequences of the WSM-associated viruses (WSMV, TriMV and HPWMoV) found in this study were utilized in a comprehensive evolutionary study of WSM viruses recently published by our group [46]. BYDV and CYDV, other economically important wheat viruses and causal agents of the barley yellow dwarf disease (BYD) complex, were also among the known wheat viruses identified in this study. In this study, we reported ~7% and 3% infections for BYDV and CYDV, respectively. In a 2008 survey in Kansas, the BYDV and CYDV infection were at 6% and 2%, respectively [47]. However, in a 2011–2012 survey, 32% of the samples were infected with BYDV and 2% with CYDV [3]. In contrast to the Kansas surveys, a survey of wheat viruses in Ohio has seen large numbers of infection of BYDV (67%) and CYDV (69%) in 2016–2017 seasons [2]. One possible explanation for these high rates of viral infections can be the large population size of the aphid vectors (*R. padi* and other species) in the surveyed fields.

In addition to known and documented wheat viruses, novel plant and fungal-associated viral sequences were also discovered in this study. A putative umbra-like viral sequence was described here. Phylogenetic analysis of the RdRp placed this new umbra-like sequence tentatively named wheat umbra-like virus (WULV) in a well-supported clade that includes previously umbra-like associated RNAs (ulaRNAs) identified in strawberry, papaya, citrus, babaco, maize, and sugarcane [48,49,50,51,52]. The sequence homology of the RdRp between WULV and other ulaRNAs ranged from 40–65% and 45–60% at the nucleotide and amino acid levels, respectively, suggesting WULV is a novel member of ulaRNAs. ulaRNAs are a group of subviral RNAs with high RdRp sequence homology to umbraviruses belonging to the genus *Umbravirus*, family *Tombusviridae* [53,54]. Umbraviruses encode their own RdRp and replication-required protein along with proteins required for cell-to-cell and long-distance movements, but they are dependent on their helper viruses, mainly from the family *Luteoviridae* for encapsidation and transmission [54].

Although the RdRp of ulaRNAs is closely related to umbraviruses and like umbraviruses, most ulaRNAs generate their own RdRp through -1 ribosome frameshift recoding [48,49,55]; their genome size and feature are different. Hence, ulaRNAs are considered as unclassified umbraviruses. Such genome size and feature variations have even been observed among ulaRNAs themselves. Liu et al. (2021) conducted a comprehensive structural analysis and genome mapping of ulaRNAs and classified this group of the coat protein-dependent RNAs into 3 classes [56].

The WULV genome organization is close to the strawberry associated virus A (SbaVA) [56]. Interestingly and in contrast to other ulaRNAs, the ORF1 and ORF2 of both SbaVA and WULV encoding replication-required proteins are not overlapped (Figure 2a). However, while SbaVA has only one ORF after RdRp with no homology to known proteins, WULV has two ORFs (Figure 2a) encoding putative proteins with no homology to proteins in the database. To the best of our knowledge, this is the first report of the complete genome sequence of an ulaRNA identified in cereals. In a recent metagenomics study, contigs illustrating sequence similarities to ulaRNAs have also been reported in Colorado wheat samples [57].

We did not identify any luteoviruses as a helper virus in our positive samples for WULV. However, our WULV infected samples were positive for WSMV which is a potyvirus. It has been also reported that the newly discovered ulaRNA in papaya (papaya umbravirus (PUV)) was always found in systematic papaya plants associated with a potyvirus, papaya ringspot virus [48,58]. Whether WSMV plays the role of the helper virus for WULV is not currently clear. Additionally, the pathogenicity of WULV in wheat needs further investigation.

The two poty-like and virga-like viral sequences were always detected together in each positive RNA sample and shared high similarity (85%) of nucleotide sequences at the 5′ UTR suggesting that these viral sequences are two segments of a new putative bi-partite virus. We tentatively named this putative virus wheat-associated vipovirus (WaVPV), as a combination of *Virgaviridae* and *Potyviridae*, the families with the greatest similarity hits for these novel viral sequences. RT-PCR without the reverse transcriptase enzyme was utilized to verify that WaVPV segments were not integrated into the host genome (data not shown). The schematic genome organization of WaVPV is similar to the recently reported 4 unclassified tobamo-like, fungal-associated viruses: plasmopara viticola lesion associated vivirus (PvLaVivivirus1–4) [59]. As with PvLaVivivirus1–4, WaVPV RNA1 and RNA2 also encode RdRp and helicase, respectively. However, WaVPV does not contain a conserved domain for methyltransferase, which has been observed to be encoded by either RNA1 or RNA2 of the PvLaVivivirus1–4 [59]. Additionally, and unlike the previous studies of unclassified fungal-associated tobamo-like viruses, WaVPV RNA2 contains the DEAD-like helicase conserved domain sharing similarities to only potyviruses infecting plants and had no significant similarity hits for tobamo-like viruses. Hence, and from an evolutionary standpoint, WaVPV is an interesting novel putative virus showing similarity to both plant and fungal viruses. Our evidence at this time is not enough to conclude that wheat is the primary or alternative host for WaVPV, and still its association with fungal hosts infecting wheat cannot be ruled out.

The evolutionary relationship between fungal and plant viruses has yet to be fully understood and presents complex and conflicting standpoints. Although the close relationship and interaction of plant and fungal-associated viruses are still being studied, some breakthroughs contain evidence to support the possibility of cross-kingdom infections. Previous studies have observed that some fungal-associated viruses can replicate in plant protoplasts without the introduction of changes to the viral genome to adapt to the plant host [60]. On the other hand, a plant virus, cucumber mosaic virus, was observed to be able to infect a fungal host, *Rhizoctonia solani*, efficiently [61]. Interestingly, a recent study has shown that a mixed-infection of a fungal-associated virus with a plant virus can aid in the replication of the plant virus in the fungal host and for the fungal-associated virus, the mixed infection aids in systemic infection of the plant host and infection of vegetative incompatible fungi of a different fungal species [62]. Hence, cross-kingdom infections of plant and fungal-associated viruses bring more questions about the origins of plant and fungal viruses.

Metagenomics and phylogenetic analyses have introduced different concepts to understand the evolutionary history of plant and fungal-associated viruses. In this study, the phylogenetic analysis of the WaVPV RNA1 based on the RdRp amino acid sequence placed the segment in the sub-clade containing both recently discovered unclassified fungal-associated and insect-specific viruses from the *Virgaviridae* family, whereas the WaVPV RNA2 did not have any fungal-associated virus similarity hits and was found to be in a close relationship with barley mild mosaic virus (BaMMV) and other plant viruses belonging to the *Potyviridae* family. These results suggest that each segment of WaVPV was obtained from viruses infecting different kingdoms and may have been a result of genetic exchange between distinct plant and fungal-associated viruses.

Reassortment is a type of genetic exchange and one of the main evolutionary mechanisms utilized by segmented RNA viruses [63]. During mixed-infections of multiple viruses, RNA segments may be exchanged between different viruses to produce novel viruses [64]. The limitation of reassortment has been accepted to be between only viruses with segmented RNAs and these segmented RNA viruses must be closely related enough to be of the same viral species; however, there have been reports of inter-kingdom genetic exchange and reassortment between different viral families leading to the creation of novel viruses [59,65]. The first report of a novel virus origin through inter-kingdom genetic exchange was in the discovery and characterization of a plant virus, ourmia melon virus (OuMV) [65]. OuMV contained three viral segments: the RdRp containing homologues of fungal viruses from *Narnaviridae* family, the movement protein (MP) containing similarities with plant viruses from the *Tombusviridae* family, and the coat protein (CP) showing close relationships with viruses infecting plants and animals [65]. Another study conducted to characterize plant viruses in grapevine samples has also discovered a novel virus, grapevine associated jivivirus, which has been suggested to be the result from the reassortment of virga-like and flavi-like viruses [59]. This suggested that reassortment may not only occur between different viral families, but also viruses infecting hosts of two different kingdoms. Similar to the latter study, the discovery of WaVPV in this study also supports the possibility of inter-kingdom genetic exchange.

In addition to known and novel plant viral sequences, known and novel fungal-associated viral sequences were also found in this study. A novel fungal-associated tobamo-like viral sequence tentatively named wheat associated tobamo-like virus (WaTBLV) was identified in this study. The organization of the near complete genome of WaTBLV was similar to tobamoviruses belonging to the family *Virgaviridae* [66]. Our phylogenetic tree built based on the RdRp amino acid sequences placed WaTBLV in a well-supported clade with other recently reported fungal- and insect-associated tobamo-like viruses and separately from plant-associated tobamoviruses (Figure 4b). This result suggests that WaTBLV is probably a fungal-associated virus with the currently unknown fungal host.

As similar to plant tobamoviruses, the ORF1 and ORF2 of newly identified tobamo-like viruses in fungi and insects contain methyltransferase, viral helicase, and RdRp conserved domains, respectively [66]. However, the ORF3 of tobamo-like viruses such as WaTBLV discovered here has a DEAD-like helicase domain, while no conserved domain has been identified for the ORF4 [31,67]. The genome size and organization of WaTBLV was close to the recently identified tobamo-like virus, macrophomina phaseolina tobamo-like virus [67]. It will also be interesting to study the potential of WaTBLV as a new biological agent to control wheat pathogenic fungi.

A new putative mitovirus was also identified in our wheat samples. Mitoviruses belong to the family *Narnaviridae* [68]. This group of viruses are considered as the simplest out of all mycoviruses, containing a positive-sense, single-stranded RNA genome which encodes only the RdRp and strictly replicate in the mitochondria of the host [68]. The low similarity (≤40%) at the amino acid level between the mitovirus identified in this study and other reported mitoviruses indicates that this may be a novel mitovirus. Although the natural fungal host of this new putative mitovirus tentatively named *Mitovirus* sp. is not yet known, our phylogenetic analysis groups it with mitoviruses infecting rust (Figure 5b) suggesting that the potential host may also be rust fungi infecting wheat. The potential of this new mitovirus as a new biological agent is worth investigating.

During replication of RNA viruses, the synthesis of the double-strand RNA (dsRNA), an intermediate replication form, triggers the antiviral defense mechanism (RNAi) of the host which leads to the degradation of RNAs into small RNAs called viral small interfering RNAs (vsiRNA) [69,70]. For plant viruses, vsiRNAs range from 21–24 nt with the peak at 21 nt while this remains unclear for fungal-associated viruses; however, multiple small RNA sequencing studies have observed the range of 20 to 24 nt [71,72,73,74]. Here, the size distributions of vsiRNAs, which resulted from mapping of small RNAs generated from an infected wheat sample against the full genomes of WaVPV and WaTBLV, had a prominent peak at 21 nt (Figure 6a–c). These results suggest that these putative viruses may originate from replicative viruses and may be processed by the antiviral RNAi machinery.

Taken together, the findings of this study emphasize the importance of identifying and characterizing all viral communities associated with wheat. However, the geographic area of sampling of this study was small. Moreover, there are still many open questions regarding the newly identified putative viruses including WULV and WaVPV, and further experiments are needed to fully characterize these two putative viruses. Studying the pathogenicity and distribution of WULV in wheat fields is crucial. Furthermore, the biology of these putative novel viruses needs to be studied. In fact, these aspects are the focus of our current research. Additionally, it is important to determine the natural host of WaVPV and whether this putative virus is a serious threat to the wheat industry.

## Figures and Tables

**Table 1:** Identified hits by BLASTx searches using the de-novo assembled contigs.

Novel Viral Sequence	Positive Libraries	Known Virus Hits	Similarity (%)	Query Coverage (%)	E Value
Umbra-like viruses	GL01_19, GL20	Unclassified umbraviruses	≤60%	<55%	<0.00001
Virga-like	NS02_19, FO19, EL19, KW19, GL01_19, ME19	Plant-associated virga viruses	≤26%	<27%	<0.00001
		Fungal-associated virga viruses	≤30%	<40%	<0.00001
Poty-like	NS02_19, FO19, EL19, KW19, GL01_19, ME19	Potyviruses	≤25%	>40%	<0.00001
Tobamo-like	NS02_19, FO19, EL19, KW19, GL01_19, ME19	Fungal-associated tobamo-like viruses and plant tobamoviruses	≤40%	~50%	<0.00001

## Data Availability

Generated reads are publicly available at NCBI Sequence Read Archive (SRA) under the BioProject number PRJNA722004.

## Supplementary Materials

The following are available online at www.mdpi.com/article/10.3390/v13122457/s1, Table S1: List of samples in this study. Table S2: List of designed primers for RT-PCR and RACE. Table S3: Results from RNASeq Analysis. Table S4: List of sequences and accession numbers used as references for mapping. Table S5: The list of sequences obtained from this study along with the accession numbers. Figure S1: Validation of viral sequences in the original RNAs. Figure S2: The alignment of the 5′ and 3′ UTRs of WaVPV RNA1 and RNA2.

## References

[1] Nelson G.C., Rosegrant M.W., Palazzo A., Gray I., Ingersoll C., Robertson R.D., Tokgoz S., Zhu T., Sulser T.B., Ringler C. (2010). Food Security, Farming, and Climate Change to 2050: Scenarios.

[2] Hodge B.A., Paul P.A., Stewart L.R. (2020). Occurrence and High-Throughput Sequencing of Viruses in Ohio Wheat. Plant Dis..

[3] Rotenberg D., Bockus W.W., Whitfield A.E., Hervey K., Baker K.D., Ou Z., Laney A.G., De Wolf E.D., Appel J.A. (2016). Occurrence of Viruses and Associated Grain Yields of Paired Symptomatic and Nonsymptomatic Tillers in Kansas Winter Wheat Fields. Phytopathology.

[4] Singh K., Jarošová J., Fousek J., Chen H., Kundu J.K. (2020). Virome Identification in Wheat in the Czech Republic Using Small RNA Deep Sequencing. J. Integr. Agric..

[5] Hollandbeck G., DeWolf E., Todd T. (2017). Kansas Cooperative Plant Disease Survey Report Preliminary 2017 Kansas Wheat Disease Loss Estimates. Plant Dis. Surv. Rep..

[6] Stenger D.C., Hall J.S., Choi I.-R., French R. (1998). Phylogenetic Relationships Within the Family *Potyviridae*: Wheat Streak Mosaic Virus and Brome Streak Mosaic Virus Are Not Members of the Genus *Rymovirus*. Phytopathology.

[7] Seifers D.L., Martin T.J., Harvey T.L., Fellers J.P., Stack J.P., Ryba-White M., Haber S., Krokhin O., Spicer V., Lovat N. (2008). Triticum Mosaic Virus: A New Virus Isolated from Wheat in Kansas. Plant Dis..

[8] Fellers J.P., Seifers D., Ryba-White M., Joe Martin T. (2009). The Complete Genome Sequence of Triticum Mosaic Virus, a New Wheat-Infecting Virus of the High Plains. Arch. Virol..

[9] Tatineni S., McMechan A.J., Wosula E.N., Wegulo S.N., Graybosch R.A., French R., Hein G.L. (2014). An Eriophyid Mite-Transmitted Plant Virus Contains Eight Genomic RNA Segments with Unusual Heterogeneity in the Nucleocapsid Protein. J. Virol..

[10] Tatineni S., Hein G.L. (2018). Genetics and Mechanisms Underlying Transmission of Wheat Streak Mosaic Virus by the Wheat Curl Mite. Curr. Opin. Virol..

[11] Miller W.A., Rasochová L. (1997). Barley Yellow Dwarf Viruses. Annu. Rev. Phytopathol..

[12] Walls J., Rajotte E., Rosa C. (2019). The Past, Present, and Future of Barley Yellow Dwarf Management. Agriculture.

[13] Bockus W.W., Appel J.A., Bowden R.L., Fritz A.K., Gill B.S., Martin T.J., Sears R.G., Seifers D.L., Brown-Guedira G.L., Eversmeyer M.G. (2001). Success Stories: Breeding for Wheat Disease Resistance in Kansas. Plant Dis..

[14] Appel J., DeWolf E., Bockus W., Todd T. (2014). Preliminary 2014 Kansas Wheat Disease Loss Estimates. Kansas Cooperative. Plant Dis. Surv. Rep..

[15] Gaunce G.M., Bockus W.W. (2015). Estimating Yield Losses Due to Barley Yellow Dwarf on Winter Wheat in Kansas Using Disease Phenotypic Data. Plant. Health Prog..

[16] Zhang T., Wang Z., Hu H., Chen Z., Liu P., Gao S., Zhang F., He L., Jin P., Xu M. (2021). Transcriptome-Wide N6-Methyladenosine (M6A) Profiling of Susceptible and Resistant Wheat Varieties Reveals the Involvement of Variety-Specific M6A Modification Involved in Virus-Host Interaction Pathways. Front. Microbiol..

[17] Nygren J., Shad N., Kvarnheden A., Westerbergh A. (2015). Variation in Susceptibility to Wheat Dwarf Virus among Wild and Domesticated Wheat. PLoS ONE.

[18] Monier A., Claverie J.-M., Ogata H. (2008). Taxonomic Distribution of Large DNA Viruses in the Sea. Genome Biol..

[19] Shi M., Lin X.-D., Tian J.-H., Chen L.-J., Chen X., Li C.-X., Qin X.-C., Li J., Cao J.-P., Eden J.-S. (2016). Redefining the Invertebrate RNA Virosphere. Nat. Cell Biol..

[20] Shi M., Lin X.-D., Chen X., Tian J.-H., Chen L.-J., Li K., Wang W., Eden J.-S., Shen J.-J., Liu L. (2018). The Evolutionary History of Vertebrate RNA Viruses. Nat. Cell Biol..

[21] Creager A.N.H., Scholthof K.-B.G., Citovsky V., Scholthof H.B. (1999). Tobacco Mosaic Virus: Pioneering Research for a Century. Plant Cell.

[22] Jeong J.-J., Ju H.-J., Noh J. (2014). A Review of Detection Methods for the Plant Viruses. Res. Plant Dis..

[23] Zakrzewski M., Rašić G., Darbro J., Krause L., Poo Y.S., Filipović I., Parry R., Asgari S., Devine G., Suhrbier A. (2018). Mapping the Virome in Wild-Caught Aedes Aegypti from Cairns and Bangkok. Sci. Rep..

[24] Murugan M., Cardona P.S., Duraimurugan P., Whitfield A.E., Schneweis D., Starkey S., Smith C.M. (2011). Wheat Curl Mite Resistance: Interactions of Mite Feeding with Wheat Streak Mosaic Virus Infection. J. Econ. Èntom..

[25] Niu J., Li X.-L., Wu Y.-L., Sun Q.-Z., Zhang W., Cao M., Wang J.-J. (2020). RNA Virome Screening in Diverse but Ecologically Related Citrus Pests Reveals Potential Virus-Host Interactions. J. Invertebr. Pathol..

[26] Tatineni S., Alexander J., Gupta A.K., French R. (2019). Asymmetry in Synergistic Interaction Between Wheat Streak Mosaic Virus and Triticum Mosaic Virus in Wheat. Mol. Plant-Microbe Interact..

[27] Roossinck M.J., Martin D.P., Roumagnac P. (2015). Plant Virus Metagenomics: Advances in Virus Discovery. Phytopathology.

[28] Mumo N.N., Mamati G.E., Ateka E.M., Rimberia F.K., Asudi G.O., Boykin L.M., Machuka E.M., Njuguna J.N., Pelle R., Stomeo F. (2020). Metagenomic Analysis of Plant Viruses Associated With Papaya Ringspot Disease in Carica Papaya L. in Kenya. Front. Microbiol..

[29] Adams I.P., Glover R.H., Monger W.A., Mumford R., Jackeviciene E., Navalinskiene M., Samuitiene M., Boonham N. (2009). Next-Generation Sequencing and Metagenomic Analysis: A Universal Diagnostic Tool in Plant Virology. Mol. Plant Pathol..

[30] Villamor D.E.V., Ho T., Al Rwahnih M., Martin R.R., Tzanetakis I.E. (2019). High Throughput Sequencing For Plant Virus Detection and Discovery. Phytopathology.

[31] Chiapello M., Rodríguez-Romero J., Ayllón M.A., Turina M. (2020). Analysis of the Virome Associated to Grapevine Downy Mildew Lesions Reveals New Mycovirus Lineages. Virus Evol..

[32] Marzano S.-Y.L., Nelson B.D., Ajayi-Oyetunde O., Bradley C.A., Hughes T.J., Hartman G.L., Eastburn D.M., Domier L.L. (2016). Identification of Diverse Mycoviruses through Metatranscriptomics Characterization of the Viromes of Five Major Fungal Plant Pathogens. J. Virol..

[33] Otuka A. (2013). Migration of Rice Planthoppers and Their Vectored Re-Emerging and Novel Rice Viruses in East Asia. Front. Microbiol..

[34] Matsumura E., Coletta-Filho H., Nouri S., Falk B., Nerva L., Oliveira T., Dorta S., Machado M. (2017). Deep Sequencing Analysis of RNAs from Citrus Plants Grown in a Citrus Sudden Death-Affected Area Reveals Diverse Known and Putative Novel Viruses. Viruses.

[35] Olmedo-Velarde A., Park A.C., Sugano J., Uchida J.Y., Kawate M., Borth W.B., Hu J.S., Melzer M.J. (2019). Characterization of Ti Ringspot-Associated Virus, a Novel Emaravirus Associated with an Emerging Ringspot Disease of Cordyline Fruticosa. Plant Dis..

[36] Ramos-González P., Chabi-Jesus C., Guerra-Peraza O., Breton M., Arena G., Nunes M., Kitajima E., Machado M., Freitas-Astúa J. (2016). Phylogenetic and Molecular Variability Studies Reveal a New Genetic Clade of Citrus Leprosis Virus C. Viruses.

[37] Bolger A.M., Lohse M., Usadel B. (2014). Trimmomatic: A Flexible Trimmer for Illumina Sequence Data. Bioinformatics.

[38] Andrews S. FastQC a Quality Control Tool for High Throughput Sequence Data. http://www.bioinformatics.babraham.ac.uk/projects/fastqc/.

[39] Howe K.L., Contreras-Moreira B., De Silva N., Maslen G., Akanni W., Allen J., Alvarez-Jarreta J., Barba M., Bolser D.M., Cambell L. (2020). Ensembl Genomes 2020—Enabling Non-Vertebrate Genomic Research. Nucleic Acids Res..

[40] Dobin A., Davis C.A., Schlesinger F., Drenkow J., Zaleski C., Jha S., Batut P., Chaisson M., Gingeras T.R. (2013). STAR: Ultrafast Universal RNA-Seq Aligner. Bioinformatics.

[41] Haas B.J., Papanicolaou A., Yassour M., Grabherr M., Blood P.D., Bowden J., Couger M.B., Eccles D., Li B., Lieber M. (2013). De Novo Transcript Sequence Reconstruction from RNA-Seq Using the Trinity Platform for Reference Generation and Analysis. Nat. Protoc..

[42] Altschul S.F., Gish W., Miller W., Myers E.W., Lipman D.J. (1990). Basic Local Alignment Search Tool. J. Mol. Biol..

[43] Edgar R.C. (2004). MUSCLE: Multiple Sequence Alignment with High Accuracy and High Throughput. Nucleic Acids Res..

[44] Kumar S. (2004). MEGA3: Integrated Software for Molecular Evolutionary Genetics Analysis and Sequence Alignment. Brief. Bioinform..

[45] Guindon S., Dufayard J.-F., Lefort V., Anisimova M., Hordijk W., Gascuel O. (2010). New Algorithms and Methods to Estimate Maximum-Likelihood Phylogenies: Assessing the Performance of PhyML 3.0. Syst. Biol..

[46] Redila C.D., Phipps S., Nouri S. (2021). Full Genome Evolutionary Studies of Wheat Streak Mosaic-Associated Viruses Using High-Throughput Sequencing. Front. Microbiol..

[47] Burrows M., Franc G., Rush C., Blunt T., Ito D., Kinzer K., Olson J., O’Mara J., Price J., Tande C. (2009). Occurrence of Viruses in Wheat in the Great Plains Region, 2008. Plant Health Prog..

[48] Quito-Avila D.F., Alvarez R.A., Ibarra M.A., Martin R.R. (2015). Detection and Partial Genome Sequence of a New Umbra-like Virus of Papaya Discovered in Ecuador. Eur. J. Plant Pathol..

[49] Sá Antunes T.F., Amaral R.J.V., Ventura J.A., Godinho M.T., Amaral J.G., Souza F.O., Zerbini P.A., Zerbini F.M., Fernandes P.M.B. (2016). The DsRNA Virus Papaya Meleira Virus and an SsRNA Virus Are Associated with Papaya Sticky Disease. PLoS ONE.

[50] Cornejo-Franco J.F., Flores F., Mollov D., Quito-Avila D.F. (2021). An Umbra-Related Virus Found in Babaco (Vasconcellea × Heilbornii). Arch. Virol..

[51] Tahir M.N., Bolus S., Grinstead S.C., McFarlane S.A., Mollov D. (2021). A New Virus of the Family Tombusviridae Infecting Sugarcane. Arch. Virol..

[52] Kwon S.-J., Bodaghi S., Dang T., Gadhave K.R., Ho T., Osman F., Al Rwahnih M., Tzanetakis I.E., Simon A.E., Vidalakis G. (2021). Complete Nucleotide Sequence, Genome Organization, and Comparative Genomic Analyses of Citrus Yellow-Vein Associated Virus (CYVaV). Front. Microbiol..

[53] Syller J. (2003). Molecular and Biological Features of Umbraviruses, the Unusual Plant Viruses Lacking Genetic Information for a Capsid Protein. Physiol. Mol. Plant Pathol..

[54] Taliansky M.E., Robinson D.J. (2003). Molecular Biology of Umbraviruses: Phantom Warriors. J. Gen. Virol..

[55] Felker P., Bunch R., Russo G., Preston K., Tine J.A., Suter B., Mo X.H., Cushman J.C., Yim W.C. (2019). Biology and chemistry of an Umbravirus like 2989 bp single stranded RNA as a possible causal agent for Opuntia stunting disease (engrosamiento de cladodios)—A Review. J. Prof. Assoc. Cactus Dev..

[56] Liu J., Carino E., Bera S., Gao F., May J.P., Simon A.E. (2021). Structural Analysis and Whole Genome Mapping of a New Type of Plant Virus Subviral RNA: Umbravirus-Like Associated RNAs. Viruses.

[57] Albrecht T., White S., Layton M., Stenglein M., Haley S., Nachappa P. (2020). Ecology and Epidemiology of Wheat Curl Mite and Mite-Transmissible Viruses in Colorado and Insights into the Wheat Virome. bioRxiv.

[58] Cornejo-Franco J.F., Alvarez-Quinto R.A., Quito-Avila D.F. (2018). Transmission of the Umbra-like Papaya Virus Q in Ecuador and Its Association with Meleira-Related Viruses from Brazil. Crop. Protect..

[59] Chiapello M., Rodríguez-Romero J., Nerva L., Forgia M., Chitarra W., Ayllón M.A., Turina M. (2020). Putative New Plant Viruses Associated with *Plasmopara Viticola* -infected Grapevine Samples. Ann. Appl. Biol..

[60] Nerva L., Varese G.C., Falk B.W., Turina M. (2017). Mycoviruses of an Endophytic Fungus Can Replicate in Plant Cells: Evolutionary Implications. Sci. Rep..

[61] Andika I.B., Wei S., Cao C., Salaipeth L., Kondo H., Sun L. (2017). Phytopathogenic Fungus Hosts a Plant Virus: A Naturally Occurring Cross-Kingdom Viral Infection. Proc. Natl. Acad. Sci. USA.

[62] Bian R., Andika I.B., Pang T., Lian Z., Wei S., Niu E., Wu Y., Kondo H., Liu X., Sun L. (2020). Facilitative and Synergistic Interactions between Fungal and Plant Viruses. Proc. Natl. Acad. Sci. USA.

[63] Marshall N., Priyamvada L., Ende Z., Steel J., Lowen A.C. (2013). Influenza Virus Reassortment Occurs with High Frequency in the Absence of Segment Mismatch. PLoS Pathog..

[64] Vijaykrishna D., Holmes E.C., Joseph U., Fourment M., Su Y.C., Halpin R., Lee R.T., Deng Y.-M., Gunalan V., Lin X. (2015). The Contrasting Phylodynamics of Human Influenza B Viruses. eLife.

[65] Rastgou M., Habibi M.K., Izadpanah K., Masenga V., Milne R.G., Wolf Y.I., Koonin E.V., Turina M. (2009). Molecular Characterization of the Plant Virus Genus Ourmiavirus and Evidence of Inter-Kingdom Reassortment of Viral Genome Segments as Its Possible Route of Origin. J. Gen. Virol..

[66] Adams M.J., Antoniw J.F., Kreuze J. (2009). Virgaviridae: A New Family of Rod-Shaped Plant Viruses. Arch. Virol..

[67] Wang J., Ni Y., Liu X., Zhao H., Xiao Y., Xiao X., Li S., Liu H. (2021). Divergent RNA Viruses in Macrophomina Phaseolina Exhibit Potential as Virocontrol Agents. Virus Evol..

[68] Hillman B.I., Cai G. (2013). The Family Narnaviridae. International Review of Cytology.

[69] Baulcombe D. (2004). RNA Silencing in Plants. Nat. Cell Biol..

[70] Agrawal N., Dasaradhi P.V.N., Mohmmed A., Malhotra P., Bhatnagar R.K., Mukherjee S.K. (2003). RNA Interference: Biology, Mechanism, and Applications. Microbiol. Mol. Biol. Rev..

[71] Kuo Y.-W., Falk B.W. (2020). RNA Interference Approaches for Plant Disease Control. BioTechniques.

[72] Lee Marzano S.-Y., Neupane A., Domier L. (2018). Transcriptional and Small RNA Responses of the White Mold Fungus Sclerotinia Sclerotiorum to Infection by a Virulence-Attenuating Hypovirus. Viruses.

[73] Pooggin M.M. (2018). Small RNA-Omics for Plant Virus Identification, Virome Reconstruction, and Antiviral Defense Characterization. Front. Microbiol..

[74] Özkan S., Mohorianu I., Xu P., Dalmay T., Coutts R.H.A. (2017). Profile and Functional Analysis of Small RNAs Derived from Aspergillus Fumigatus Infected with Double-Stranded RNA Mycoviruses. BMC Genom..

