# Supplementary material for: Metagenomics Analysis of the Wheat Virome Identifies Novel Plant and Fungal-Associated Viral Sequences
Source: Viruses. 2021 Dec 7;13(12):2457. doi: 10.3390/v13122457 (PMC8705367; doi:10.3390/v13122457)
Supplement: Supplementary file 1 [file viruses-13-02457-s001.zip › viruses-1460379-Supplementary figure.pdf]

viruses

MDPI

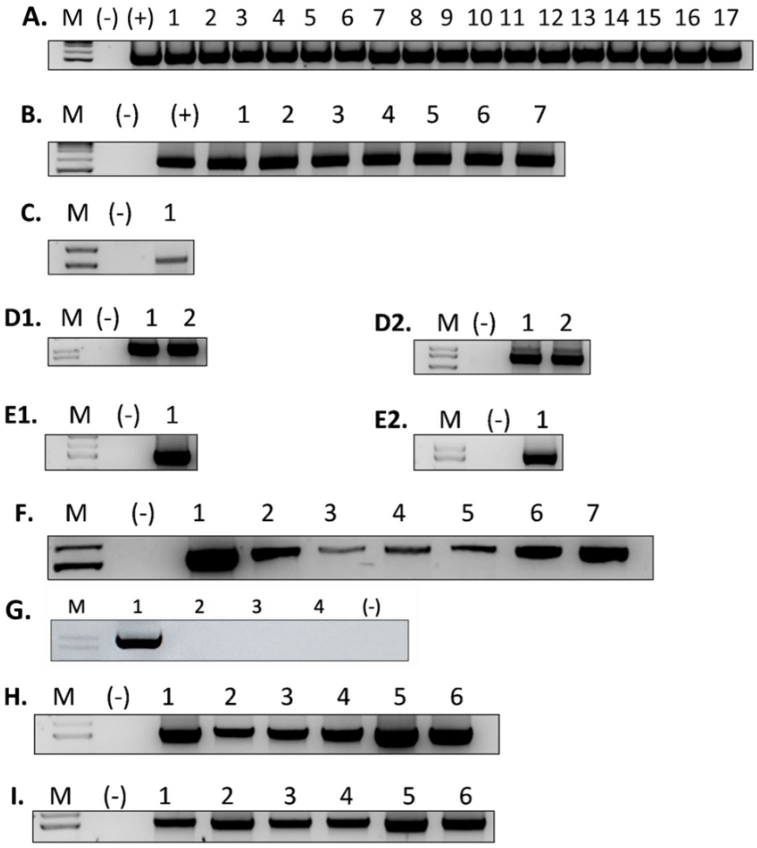

Figure S1. Validation of viral sequences in the original RNAs. M) 1Kb plus DNA ladder; A) WSMV and the samples are as follows (1-17): FO19, NS02, RP19, EL19, RA02, NE01, SM19, SH02, ST19, SA19, KM19, KW19, DC19, FW40, ME19, NT 19, and KE19; B) TriMV and the samples are as follows (1-7): EL19, NS02, RA02, DC19, NT19, SM19, and SH02; C) the only positive HPWMoV sample: RA02; D1) BYDV RdRp: KE19 and LE01; D2) BYDV CP: KE19 and LE01; E1) CYDV RdRp: EL19; E2) CYDV CP: EL19; F) *Mitovirus* sp.for the following samples (1-7): GL01_19, KM19, NT19, ME19, LE01, KW19, and SA19; G) WULV for the following (1-4): GL20, KW19, FO19, and KE19; H) WaVPV RNA1 for the following samples (1-6): GL01_19, ME19, KW19, EL19, FO19, and NS02; I) WaVPV RNA2 for the following samples (1-6): GL01_19, ME19, EL19, KW19, NS02, and FO19. RNA extracted from a healthy wheat plant (no symptoms) was used as a negative control (-) for screenings.

```
5' WaVPV RNA 2 AGTTTTTGTCCTGCGTCCTTGAGACGTGACGAAAATCGTACATTAGGTACTTCCCGACAC    60
5' WaVPV RNA 1 AGTTTTTGTCCTGCGTCCTTGAGACGTGACGAAAATCGTACTATAAGTACTTCCCGAACC    60

5' WaVPV RNA 2 CACAACAAACGAACCGACCACACACGACACGACCACAAAT-------               100
5' WaVPV RNA 1 GACCACAAACCAAAACACCACACCCGACCCGACCACACAACACCGTA               107

3' WaVPV RNA 2 GTCACCAATTATATGCAATCCCTTACCAATTTATTCT-GTCCGTTGTGGACAATATGCT     58
3' WaVPV RNA 1 GTTGCT-GTCGTTTAGTTTAGTTTATTTATTTACTTTTAGATCATTGTGATCAA------    53

3' WaVPV RNA 2 ATATGTTATGCGCGTGAGTTATCACGTATTGAAATTTCCTTAAAACACGAAAGGAG---   114
3' WaVPV RNA 1 ---------ACCTTTAAGTTGT----TATT----TTTTTCCAAAACACGAGGAGAAAG    95
```

Figure S2. The alignment of the 5′ and 3′ UTRs of WaVPV RNA1 and RNA2. The sequence alignments showed a high percentage of similarity in the 5′ end highlighted in yellow.
